# Adaptive Decision-Level Fusion for Osteosarcoma Classification Using Unpaired X-Ray and Histopathology Data

**DOI:** 10.3390/jimaging12100491

**Published:** 2026-10-06

**Authors:** Zainab Fouad, Sally Saad, Abeer M. Mahmoud, Abdel-Badeeh M. Salem

**Affiliations:** Computer Science Department, Faculty of Computer and Information Science, Ain Shams University, Cairo 11566, Egypt; sallysaad@cis.asu.edu.eg (S.S.);

**Keywords:** osteosarcoma, deep learning, classification, histopathology, radiology, late fusion

## Abstract

Osteosarcoma is an aggressive bone malignancy in children and adolescents, where early and accurate classification is essential for improving clinical outcomes. While deep learning methods have shown promising results in medical image analysis, most existing approaches rely on single-modality data or assume patient-level correspondence in multimodal learning, which is rarely available in real-world clinical settings. In addition, many fusion strategies are designed for paired datasets and depend on fixed weighting schemes, limiting their applicability to heterogeneous and independently collected data. To address these challenges, this study proposes a modality-agnostic ensemble learning framework across independent cohorts for osteosarcoma classification using histopathology and X-ray images. Each modality is first modeled independently using EfficientNet-B0, and decision-level integration is performed without requiring cross-modal alignment. Two fusion strategies are investigated: fixed-weight probability fusion and a proposed adaptive sample-level gating network that dynamically learns fusion weights based on modality-specific predictive confidence. Experiments conducted on publicly available histopathology (TCIA) and X-ray (BTXRD) datasets demonstrate that the proposed adaptive fusion approach achieves superior performance compared to fixed fusion. The adaptive gating network reaches an accuracy of 97.08%, outperforming fixed-weight fusion (94.15%) and unimodal baselines. These results confirm that adaptive decision-level fusion can effectively leverage complementary information across unpaired datasets. Overall, the proposed framework provides a practical solution for multimodal medical image classification under data fragmentation conditions, where paired datasets are not available.

## 1. Introduction

Osteosarcoma is an aggressive primary bone malignancy that predominantly affects children and adolescents. It is characterized by high metastatic potential and poor prognosis if not detected early [1,2]. Accurate classification is therefore critical for improving treatment planning and patient survival. However, in clinical practice, diagnosis remains challenging due to tumor heterogeneity and the complexity of interpreting medical imaging data across different modalities.

Traditional diagnostic workflows rely on radiological imaging such as X-ray, CT, and MRI, as well as histopathological examination [3]. While radiological images capture macroscopic structural changes in bone, histopathology provides detailed cellular-level information. Although both modalities are complementary, most existing deep-learning-based studies analyze them independently, limiting their ability to fully exploit cross-modal information [4].

Recent advances in deep learning have significantly improved performance in medical image classification tasks, particularly using convolutional neural networks and transfer learning. However, most osteosarcoma studies remain unimodal, focusing either on radiology or histopathology. Even in multimodal approaches, a major limitation is the assumption of patient-level correspondence between modalities, where paired samples are required for fusion. In real-world clinical settings, such paired datasets are rarely available, as imaging and pathology data are often collected independently across different institutions and workflows.

Furthermore, existing fusion strategies typically rely on fixed weighting schemes or feature-level alignment, which limits their ability to adapt to variability across samples and modalities. These constraints reduce their effectiveness when dealing with heterogeneous and unpaired datasets, a common scenario in practical medical environments.

To address these limitations, this study proposes a modality-agnostic ensemble learning framework across independent cohorts, where histopathology and X-ray images are processed independently and integrated only at the decision level without requiring cross-modal alignment. Within this framework, an adaptive sample-level gating mechanism is introduced to dynamically learn fusion weights based on modality-specific predictive confidence. This allows the model to adjust the contribution of each modality for individual samples.

Unlike traditional multimodal learning approaches, the proposed method does not assume paired inputs or shared patient identifiers across modalities. Instead, it models each modality independently and performs fusion in a flexible, data-driven manner. The framework is evaluated against fixed-weight fusion to quantify the benefits of adaptive decision-level integration under unpaired conditions.

Recent work has investigated decision-level fusion of unpaired radiographic and histopathological images for osteosarcoma classification. In this study, we focus on sample-adaptive probability-level fusion, where the contribution of each modality is dynamically adjusted according to the modality-specific prediction evidence. In addition to classification performance, we analyze the learned modality weights to provide insight into the contribution of the two imaging modalities. Therefore, the main contribution of this work is the formulation and analysis of adaptive probability-level fusion within the considered unpaired multimodal setting, rather than claiming novelty solely from the use of multimodal fusion.

This paper examined two late fusion techniques: fixed alpha fusion and an adaptive gating network. This work’s main contributions include:

- Modality-agnostic ensemble learning across independent cohorts:
  The study proposes a fusion framework that integrates histopathology and X-ray modalities without requiring patient-level correspondence, addressing a common limitation in real-world medical datasets where multimodal alignment is infeasible.
- Adaptive sample-level gating fusion for unpaired medical data:
  A lightweight gating network is introduced to learn dynamic, sample-specific fusion weights based on modality prediction distributions, enabling adaptive decision-level integration under heterogeneous data conditions.
- Systematic comparison of fusion strategies under unpaired constraints:
  The study evaluates fixed-weight fusion versus adaptive gating under identical training conditions.
- Evaluation of unimodal baselines (X-ray-only and histopathology-only models) to quantify the contribution of each modality and demonstrate the effectiveness of fusion compared to single-modality classification.

In this study, the term “adaptive” refers to sample-level adaptive fusion rather than patient-specific clinical adaptation. Specifically, the proposed gating network learns dynamic fusion weights based on the input probability distributions of each modality, allowing the contribution of X-ray and histopathology predictions to vary across individual samples.

The organization of this research is as follows: Firstly, related work is presented in Section 2. The proposed method is then discussed in Section 3. Then, the results and discussion are presented in Section 4 and Section 5. After that, the conclusion and future work are outlined in Section 6.

## 2. Related Work

X-rays remain the primary classification tool for osteosarcoma due to affordability and accessibility. Shao et al. [5] developed a deep learning model using X-ray images, focusing on distinguishing giant cell tumors from osteolytic osteosarcoma. Similarly, Wang et al. [6] proposed an ensemble deep learning framework combining multicenter radiographs to improve classification performance. However, both studies rely solely on X-rays, representing a single-modality approach, and lack sample-adaptive strategies. Wang et al.’s ensemble, while comprehensive, also introduces computational complexity that may limit practical deployment.

Histopathology remains the gold standard for osteosarcoma classification. Borji et al. [7] used a hybrid CNN-ViT model to classify osteosarcoma slides into four categories. Despite high patch-level accuracies, patient-level predictions were not explicitly validated, and slide-level data leakage may affect generalizability. Moreover, ensemble and hybrid models often involve high computational cost, limiting clinical applicability.

Prior multimodal approaches in bone tumor analysis, including CT/MRI fusion and histopathology integration, were explored. Du et al. [8] used AI-assisted CT and MRI fusion for 3D tumor margin assessment, while Huo et al. [9] combined CT and MRI with histology through a deep learning framework. Both approaches rely on fixed fusion strategies and are not adaptive to patient-specific differences, which can limit performance in heterogeneous datasets.

Adaptive fusion techniques have been explored in other cancers. Li et al. [10] presented an adaptive multimodal framework combining dermoscopy images, clinical images, and metadata for skin lesions, demonstrating the advantage of sample-adaptive weighting. Chen et al. [11] proposed a dual-view adaptive MRI fusion technique for prostate cancer grading. While promising, these methods have not been validated or optimized for osteosarcoma, highlighting the need for disease-specific adaptive approaches.

Recent studies have further advanced deep-learning-based cancer classification using both transformer and EfficientNet architectures. Mahanty et al. in [12] proposed an explainable Vision Transformer framework for multi-class oral cancer histopathology classification, combining convolutional and transformer representations with explainability mechanisms. Nallamalla and Mahanty in [13] investigated EfficientNet-B3 with a hybrid loss combining weighted cross-entropy and focal loss to address class imbalance and difficult histopathology samples. These studies demonstrate the value of improved representation learning, explainability, and loss design for cancer image analysis. In contrast, the present work focuses on a different methodological problem: adaptive decision-level fusion of independently collected X-ray and histopathology cohorts without patient-level correspondence. The contribution therefore lies primarily in the probability-level adaptive weighting mechanism rather than in proposing a new image backbone. Malik et al. [14] proposed a unified multimodal Transformer framework for breast cancer recurrence prediction and survival analysis, employing cross-modal feature fusion and attention mechanisms to integrate heterogeneous clinical and molecular information, demonstrating the potential of adaptive multimodal learning for patient-level cancer prediction. However, the framework was developed for breast cancer and did not investigate radiology–histopathology fusion or osteosarcoma, leaving its applicability to osteosarcoma diagnosis unexplored. Table 1 summarizes the related work section with limitations for each method.

Despite significant progress in single-modality and multimodal deep learning approaches for osteosarcoma classification, several important limitations remain unresolved. From a data perspective, most existing multimodal methods assume paired patient-level correspondence between imaging modalities, which is rarely available in real clinical practice, where radiology and histopathology data are typically collected independently. From a methodological perspective, current fusion strategies are largely based on either feature-level integration or fixed-weight decision fusion, which do not adapt to sample-specific variability and therefore limit the effective exploitation of complementary information across modalities. Moreover, existing adaptive fusion methods in the literature still rely on aligned multimodal representations, unlike the proposed independent cohort design that does not require modality pairing or feature-level correspondence. From a clinical generalization perspective, many proposed models are evaluated under single-center or limited dataset conditions, with insufficient validation across institutions, imaging protocols, and population distributions, raising concerns about robustness under domain shift. These limitations highlight the need for a modality-agnostic and adaptive fusion framework capable of operating under unpaired and heterogeneous data conditions, which motivates the proposed approach in this study.

## 3. Proposed Method

### 3.1. Base Model

The framework combines two modalities, one trained on X-ray images and another on histopathology images.

**Figure 1:**
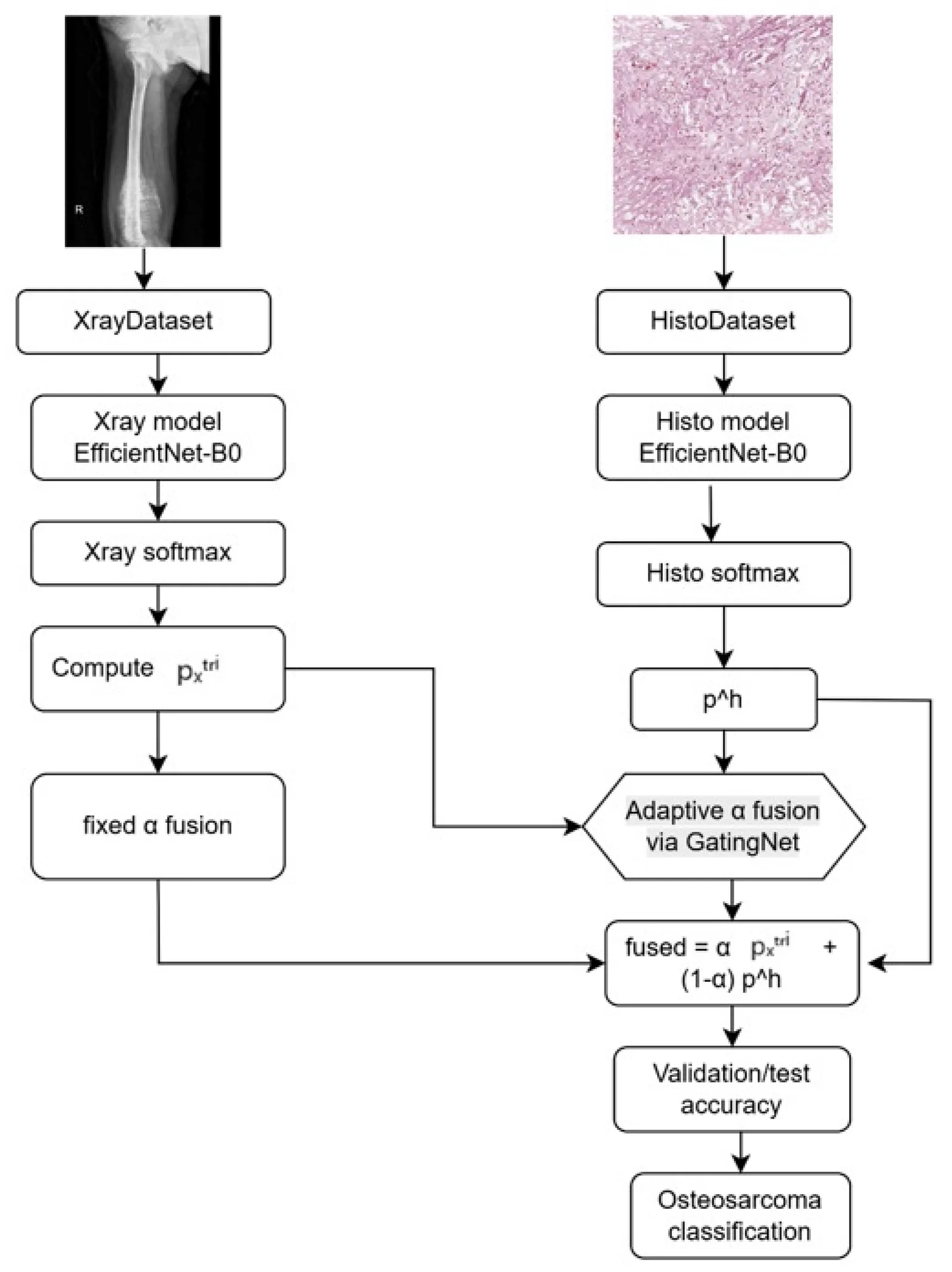
Overview of the proposed framework for osteosarcoma analysis. X-ray and histopathology images are processed independently with EfficientNet-B0, producing probability vectors. Two fusion strategies are applied at the probability level. **Fixed α Fusion**: a global weight α is used to combine the two modalities. **Adaptive Gating Network Fusion**: a lightweight gating network dynamically learns sample-specific weights from the modality probabilities, enabling adaptive late fusion.

Each module used an EfficientNet-B0 pretrained on ImageNet. The two datasets were split into training, validation, and testing sets. X-ray and histopathology images are processed independently with EfficientNet-B0, producing a feature vector. Each module was fine-tuned with a three-class classification head. Training employed the Adam optimizer with cross-entropy loss. Figure 1 shows the proposed framework. The training hyperparameters were evaluated over a small candidate set, and the selected configuration was used for the final experiments. The evaluated and selected values are summarized in Table 2.

Each modality is evaluated independently on its respective dataset using its corresponding ground-truth labels. During inference, fusion is performed at the decision level by combining the predicted probability distributions from X-ray and histopathology models. Since the X-ray and histopathology datasets originate from independent cohorts, no patient-level correspondence exists between the two modalities. Therefore, the proposed framework does not perform patient-level multimodal fusion. Instead, each modality is processed independently to generate probability distributions, and these predictions are combined at the probability level to investigate adaptive modality weighting. The fusion therefore represents probability-level fusion of independently generated modality predictions rather than the combination of corresponding X-ray and histopathology images from the same patient.

Parameters used for model training. EfficientNet-B0 was selected for its balance between accuracy and computational efficiency, providing reproducible results on limited GPU resources. A dropout rate of 0.5 was chosen to reduce overfitting while maintaining sufficient learning capacity. The learning rate of 1 × 10^−3^ offers stable training and efficient convergence, balancing speed and stability.

### 3.2. Dataset and Preprocessing

This study utilized two separate datasets: the X-ray dataset from BTXRD [15] and the histology dataset from the Osteosarcoma-Tumor-Assessment collection (TCIA) [16].

The TCIA histopathology dataset contained 536 non-tumor images, 263 non-viable tumor, and 345 viable tumors. The BTXRD radiograph dataset included 297 osteosarcoma images and 300 normal images, selected to maintain class balance. For the histopathology dataset, splitting was performed at the WSI level to ensure all patches derived from the same slide remained within a single partition. For the X-ray dataset, splitting was performed using center-wise grouping to separate acquisition centers across partitions and reduce center-specific bias.

Since the histopathology (TCIA) and X-ray (BTXRD) datasets originate from independent cohorts, no one-to-one correspondence exists between samples. Therefore, the fusion process does not assume paired patient-level alignment. Instead, both modalities are independently processed, and fusion is performed at the probability level using modality-agnostic representations.

**Figure 2:**
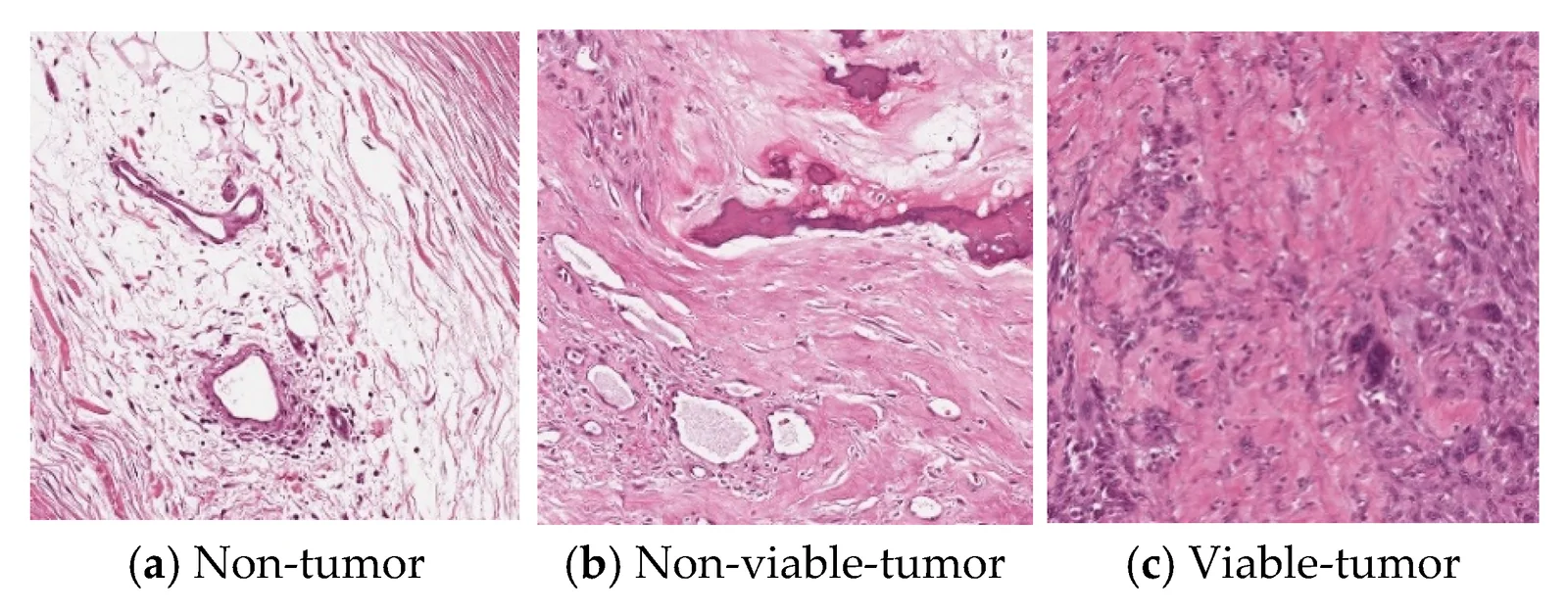
Osteosarcoma-Tumor-Assessment (TCIA) Dataset Samples [16]. (**a**) Non-tumor, (**b**) Non-viable-tumor and (**c**) Viable-tumor.

**Figure 3:**
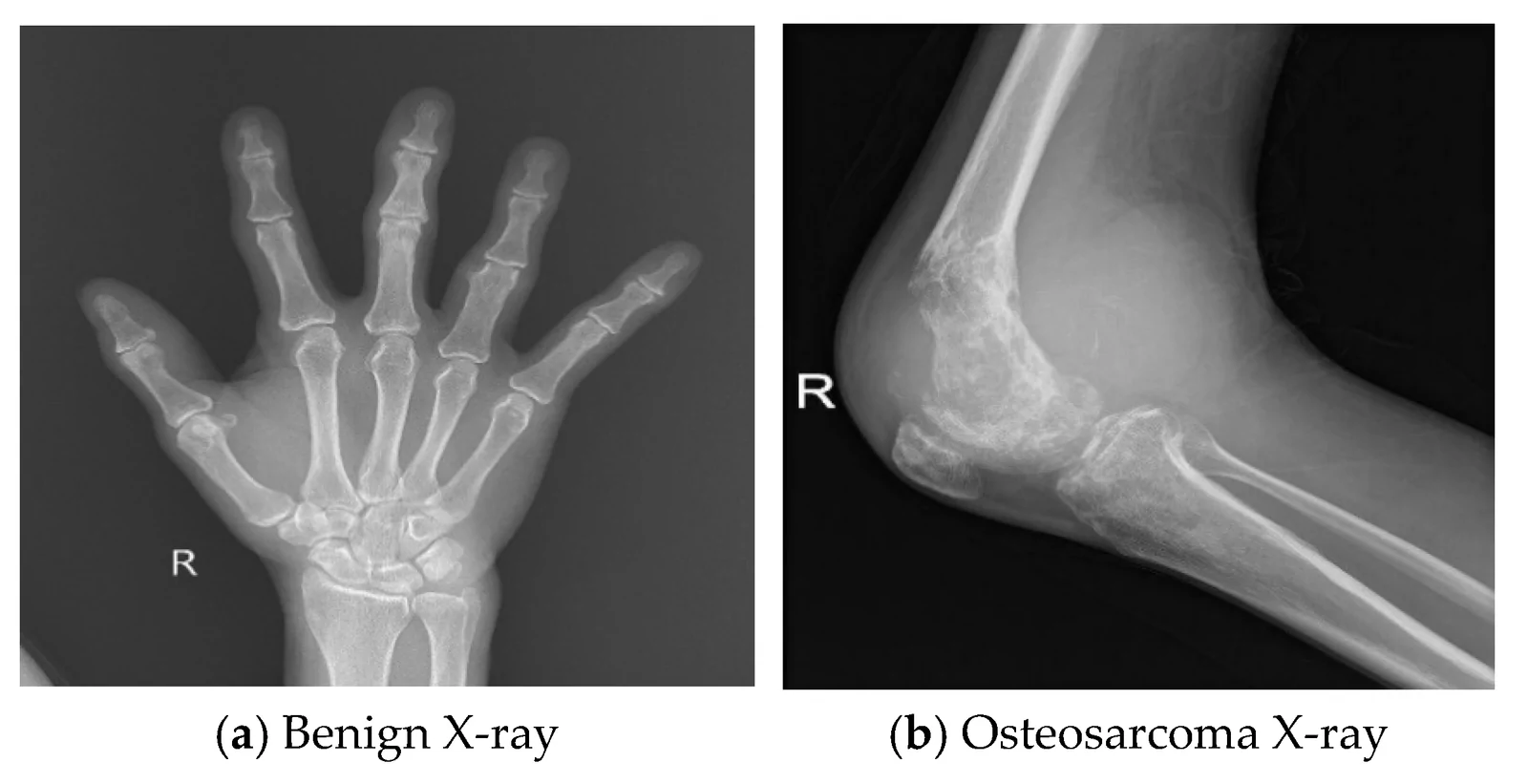
BTXRD dataset samples [15]. (**a**) Benign X-ray and (**b**) Osteosarcoma X-ray. Note: R refers to the right side.

Representative samples from these datasets are displayed in Figure 2 and Figure 3. To ensure uniform input to the models, all images were scaled to 224 × 224 pixels. Data augmentation was used to enhance model generalization: X-ray images were augmented with contrast modifications, horizontal flips, and random cropping, while histopathological images were subjected to random rotations, horizontal and vertical flips, and color jitter.

The BTXRD X-ray classifier was evaluated independently as a binary normal-versus-osteosarcoma task, whereas the histopathology and fusion models were evaluated on the three-class TCIA task; therefore, the X-ray and fusion results are reported separately and do not represent patient-level paired multimodal predictions.

### 3.3. Fixed Fusion

In fixed alpha fusion, the histopathology and X-ray predictions are combined using a constant weight α ∈ [0,1] via Equations (1) and (2). The pseudocode of fixed fusion is shown in Algorithm 1 [17].

p_x_ᵗʳⁱ = [ p_tumor . h_viable/(h_viable + h_nonviable), p_tumor . h_nonviable/(h_viable + h_nonviable), p_benign ] (1)

For example, suppose:

X-ray: p_tumor = 0.80

X-ray p_benign = 0.20

Histopathology: h_viable = 0.60

h_nonviable = 0.30

h_nontumor = 0.10

Then:

p_x_ᵗʳⁱ = [0.80 . {0.60/0.60 + 0.30}, 0.80 . {0.30/0.60 + 0.30}, 0.20]

giving: p_x_ᵗʳⁱ =[0.5333,0.2667,0.2000].

Because the X-ray classifier produces binary tumor/benign probabilities, the tumor probability is divided into viable and non-viable tumor components using the relative viable and non-viable probabilities predicted by the histopathology classifier. Consequently, the resulting three-class X-ray representation is not completely independent of the histopathology prediction. This transformation is used to represent both modalities in the same three-class probability space before fusion [17].

p_fused = α · p_x_ᵗʳⁱ + (1 − α) · p_h (2)

Variable Notes:

- p_tumor and p_benign are the softmax probabilities from the X-ray classifier.
- h_viable and h_nonviable are the tumor-related histopathology probabilities.
- p_x_ᵗʳⁱ is the tri-class converted X-ray probability distribution.
- p_h = [h_viable, h_nonviable, h_nontumor] are the histopathology softmax probabilities.
- α is the fusion weight controlling the contribution of each modality; it is fixed (e.g., 0.0, 0.25, 0.5, 0.75, 1.0).
- p_fused fused class-probability vector from X-ray and histopathology predictions.

**Algorithm 1:** Fixed alpha fusionInputs: X-ray model.Histopathology model.X-ray and histology data loaders.Fusion weight α ∈ [0,1]. Procedure: For each of X-ray and histology images:a.Compute X-ray probabilities using the X-ray model;b.Compute histology probabilities using the histology model.Split tumor probability according to histology subtypes.Construct tri-class X-ray probability vector combining tumor splits and benign probability.Fuse probabilities: fused=α× X-ray tri-class + (1−α)× histology.Normalize fused probabilities so they sum to 1.Predict class: pred=arg max (fused).

### 3.4. Adaptive Fusion via Gating Network

In contrast to early, mid-level, or fixed fusion strategies that assume uniform informational value across inputs, the adaptive fusion strategy determines the contribution of each modality dynamically through a lightweight gating network that predicts a sample-specific fusion coefficient α ∈ [0,1]. The network receives concatenated probability distributions from both modalities and outputs an adaptive weight for computing the final fused prediction using Equations (3)–(5). Table 3 summarizes the gating network parameters.

#### 3.4.1. Fusion Formulation

For each sample, the tri-class X-ray probability vector p_x_ᵗʳⁱand the histopathology probability vector p_h are concatenated to form the fusion input [18]:

gate_input = [ p_x_ᵗʳⁱ, p_h ]. (3)

The gating network maps this input to a continuous fusion weight [18]:

α = GatingNet(gate_input), α ∈ [0, 1].(4)

The final fused probability distribution is obtained by [18]:

p_fused = α · p_x_ᵗʳⁱ + (1 − α) · p_h (5)

Variable Notes:

- p_x_ᵗʳⁱ is the tri-class converted X-ray probability distribution.
- p_h is the histopathology softmax probabilities.
- α in this adaptive fusion is sample-dependent, learned to optimize classification performance via pseudo-labeling.
- The gating network is trained with pseudo-labels α* determined as the α that maximizes per-sample prediction accuracy over a discrete set [0.0, 0.25, 0.5, 0.75, 1.0].

#### 3.4.2. Pseudo-Label Computation (α*)

To train the gating network, pseudo-labels α* were assigned only to training samples, ensuring that no validation or test information was used at any stage.

Pseudo-label supervision was adopted to provide explicit supervision for learning sample-dependent fusion weights in the unpaired setting. Compared with direct end-to-end optimization, this approach improves training stability, reduces the risk of trivial collapse to constant modality weighting, and provides a controlled mechanism for learning adaptive fusion behavior under limited data.

For each training instance, five candidate fusion weights [0.0, 0.25, 0.5, 0.75, 1.0] were evaluated. For each candidate α, a fused prediction was computed. The pseudo-label α* was then assigned according to a hierarchical selection strategy: first, the α that yields a correct class prediction is selected; if multiple values satisfy this condition, the α producing the highest fused softmax confidence is chosen; if none of the candidates produce a correct prediction, α* is selected as the α that minimizes the classification error (as defined in Equation (6)) [18].

(6) $$\alpha^{*}=arg\;\underset{\alpha \in A}{max}\;\mathrm{Acc}(f(\alpha ))$$

Variable Notes:

- A={0.0,0.25,0.5,0.75,1.0}
- f(α) denotes the fused output under candidate α.

The candidate set ([0, 0.25, 0.5, 0.75, 1.0]) was selected as a simple and computationally controlled set of reference fusion weights for pseudo-label generation. These values are used only during pseudo-label construction; they do not restrict the final gating network, which predicts a continuous fusion coefficient (α in [0,1]). The discrete candidate set provides a reproducible approximation of the preferred modality contribution for each training sample while avoiding an exhaustive search over a fine-grained continuous range.

#### 3.4.3. Generalization and Leakage Prevention

Because pseudo-labels α* are derived exclusively from the training set using its ground-truth labels, the gating network never receives information from the validation or test sets. This ensures no label leakage, no optimization bias toward validation, and no artificial inflation of reported performance.

Dropout, L2 regularization, and early stopping further reduce the risk of overfitting, allowing the gating network to learn stable, generalizable weighting functions.

The adaptive gating network provides a rigorously defined, reproducible mechanism for learning sample-dependent fusion weights. Its controlled architecture, transparent pseudo-labeling strategy, and strict separation of training/validation/test data address all methodological considerations related to reproducibility, generalization, and fusion reliability. The pseudocode of adaptive fusion is shown in Algorithm 2.

To assess the robustness of the proposed framework to stochastic training variability, each experimental configuration was evaluated over 10 independent experimental runs. Performance is reported as mean ± standard deviation (SD) across the 10 runs. In addition, 95% confidence intervals (CIs) for the mean accuracy were calculated using Student’s t-distribution with 9 degrees of freedom. The number of independent runs and the corresponding variability estimates are reported to provide a more comprehensive assessment of model performance and robustness. Statistical comparisons between the fixed-weight fusion and the proposed adaptive fusion were performed using the run-level test accuracies, with a two-sided significance level of 0.05. The corresponding statistical results are reported together with the performance estimates.
**Algorithm 2:** Adaptive fusion with gating networkInputs: X-ray model.Histopathology model.Gating network.Data loaders. Training Procedure: For each of X-ray and histology images: a.Compute X-ray tri-class probabilities;b.Compute histology probabilities;c.Concatenate tri-class and histology probabilities as gating input.Generate pseudo-label α: ◦Test candidate α values (e.g., 0, 0.25, 0.5, 0.75, 1);◦Select α giving the best prediction accuracy for each sample.Predict α using the gating network.Compute the loss between the predicted α and pseudo-label.Update gating network parameters. Inference Procedure: For each of X-ray and histology images: a.Compute X-ray tri-class probabilities;b.Compute histology probabilities;c.Concatenate as gating input.Predict α using the gating network.Fuse probabilities: fused=α.  X-ray tri-class +(1−α). histology.Predict class: pred=arg max(fused).

## 4. Results

### 4.1. Performance of Fixed-α Fusion

Across all α values, the performance of the fixed-α fusion strategy showed clear variation, confirming the sensitivity of the model to the predetermined weight distribution. The optimal configuration was observed at α = 0.25, achieving a test accuracy of 94.15%. Training accuracy remained consistently higher than validation accuracy throughout training, indicating stable learning yet highlighting a noticeable gap in generalization.

Detailed metrics for each class—including precision, recall, F1-score, and specificity are summarized in Table 4A. All experiments were repeated multiple times with different random initializations, and results are reported as mean ± standard deviation.

### 4.2. Performance of Adaptive Gating Fusion

The adaptive gating network exceeded the performance of fixed-α configurations, reaching a test accuracy of 97.08%. The training and validation curves exhibited smoother convergence with a narrower gap, indicating superior generalization capability. The class-wise metrics (Table 4B) show a consistent improvement in precision, recall, and F1-score across all classes. All experiments were repeated multiple times with different random initializations, and results are reported as mean ± standard deviation.

### 4.3. Confusion Matrix Interpretation

Table 5 presents the three-class confusion matrices for the fixed-α and adaptive fusion strategies on the TCIA evaluation set. The BTXRD X-ray classifier was evaluated separately as a binary classifier. Because the two datasets contain independent cohorts without patient-level correspondence, these results should not be interpreted as patient-level multimodal diagnostic performance. Under the fixed-α setting, 3 non-tumor samples were misclassified, whereas the adaptive gating approach reduced these errors to 2. For viable tumors, the fixed-α model incorrectly labeled 3 cases as non-tumor, while the adaptive method reduced this misclassification to a single case. Similarly, for non-viable tumors, the fixed-α fusion produced 4 errors (two per class), compared with only 2 in the adaptive configuration. Overall, the adaptive gating fusion consistently demonstrated improved reliability by reducing misclassification rates across all tumor categories.

### 4.4. Training Behavior and Convergence

**Figure 4:**
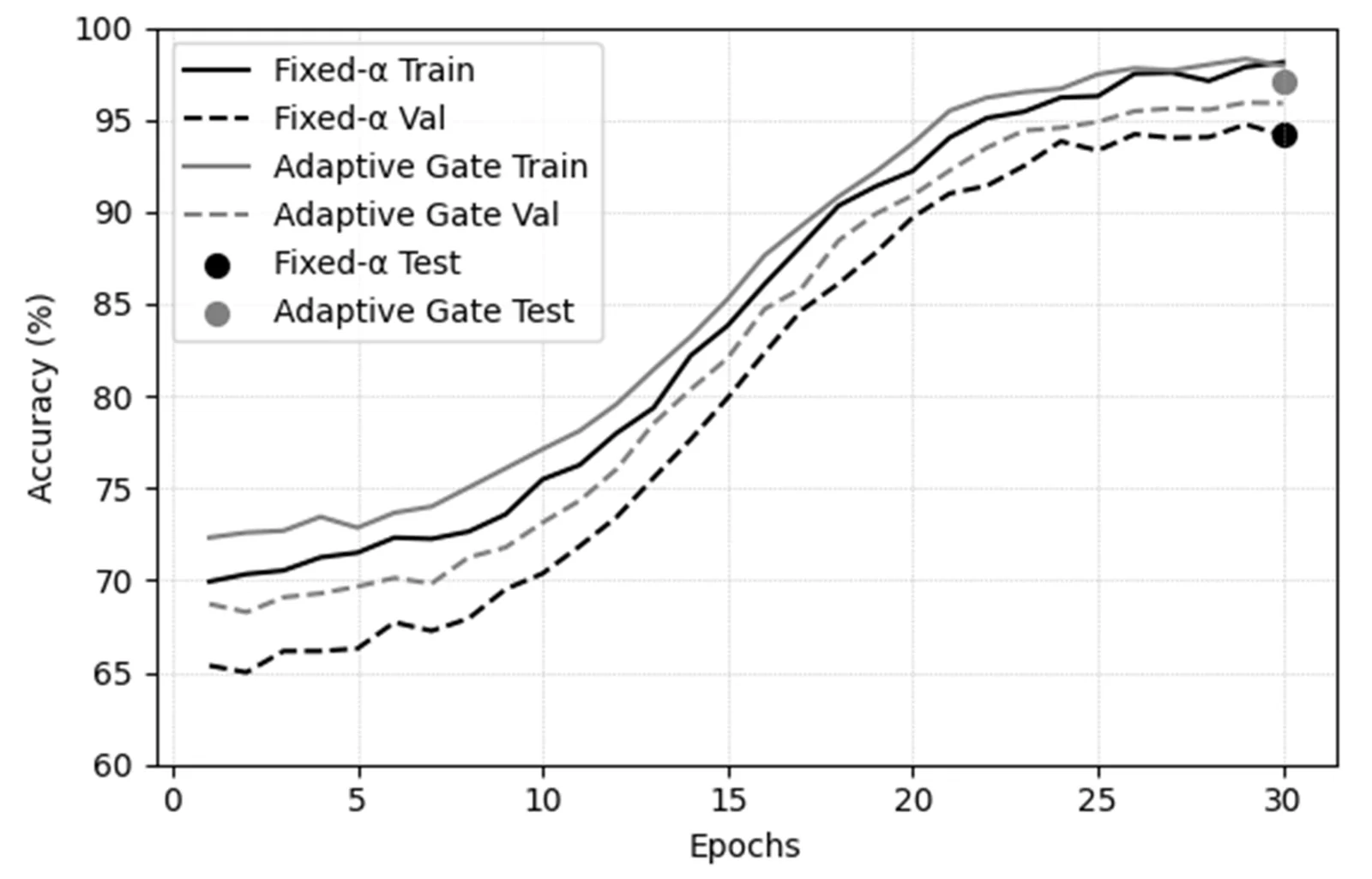
Fusion Strategy Training Curves. Training and validation accuracy curves for the fixed-α fusion and adaptive gating fusion strategies. The x-axis represents the number of epochs, and the y-axis shows accuracy (%). The legend distinguishes training, validation, and test performance for both fusion methods.

Figure 4 illustrates training and validation accuracy curves.

Fixed-α fusion shows regular convergence but retains a noticeable gap between training and validation accuracy, reflecting limited generalization. The adaptive gating fusion produces smoother, better-aligned curves, confirming its ability to dynamically adjust fusion weights and maintain stable learning across epochs.

Comparison of these results with a subset of representative methods is shown in Table 6.

A comparison of the proposed methods with existing approaches demonstrates the potential benefits of the integration. The comparison considers the closest related methods, but caution is warranted since some baselines employ different datasets, modalities, or patient cohorts. On the TCIA dataset, Anisuzzaman et al. [17] used transfer learning with pre-trained CNNs and reported 95.65% accuracy, while other unimodal CNN architectures on TCIA achieved lower accuracies, such as 86% [18] and 91% [19]. Methods incorporating non-imaging data, such as CT scans, demographics, and genetic markers [20], reached 80% accuracy, highlighting the challenges of combining heterogeneous data sources. More recent works using advanced feature extractors or deep architectures achieved higher accuracies on TCIA, including XGBoost-based frameworks [21] with an accuracy of 96.84% and VGG19 [22] with an accuracy of 96.27%. Approaches using different modalities, such as hyperspectral imaging combined with H&E-stained histopathology [24], achieved 87.33%.

The proposed fixed fusion approach (94.15%) performs competitively relative to these baselines, despite integrating radiographs and histopathology from independent datasets. The adaptive fusion method (97.08%) further improves performance by dynamically weighting contributions from each modality.

It is important to note that the accuracies reported across prior studies are not directly comparable to our results, as the datasets, imaging modalities, cohort sizes, and acquisition conditions differ substantially. Therefore, the comparative analysis in Table 6 should be interpreted only as a contextual reference rather than a standardized benchmark, highlighting that adaptive fusion can achieve accuracy comparable to or exceeding existing single- and multimodal approaches.

### 4.5. Unimodal and Fusion Model Performance Comparison

To evaluate the contribution of fusion, the unimodal baselines were first assessed independently on their respective held-out test sets using the same training and testing protocol. The X-ray-only model was evaluated on the BTXRD test set using binary normal/osteosarcoma labels and achieved an accuracy of 93.07%, while the histopathology-only model achieved 94.12% on the corresponding histopathology test set. These unimodal results are reported independently because the two datasets contain different, unpaired cohorts.

The fixed-α fusion method achieved 94.15 ± 3.55%, whereas the adaptive gating fusion achieved 97.08 ± 2.85% under the defined three-class fusion evaluation. Thus, the fusion results should be interpreted as decision-level evaluation under the unpaired-data protocol rather than as patient-level multimodal diagnostic performance.

The fixed-α fusion method showed a marginal improvement with an accuracy of 94.15 ± 3.55%, suggesting limited benefit from static fusion weighting. In contrast, the proposed adaptive gating fusion achieved the best performance with an accuracy of 97.08 ± 2.85%, outperforming both unimodal models and the fixed fusion approach.

The improvement of the adaptive fusion model exceeds the observed variability (±SD), indicating that the gain is consistent and not due to random fluctuations. This confirms that the adaptive weighting mechanism effectively leverages complementary information from both modalities, rather than simply combining them.

**Figure 5:**
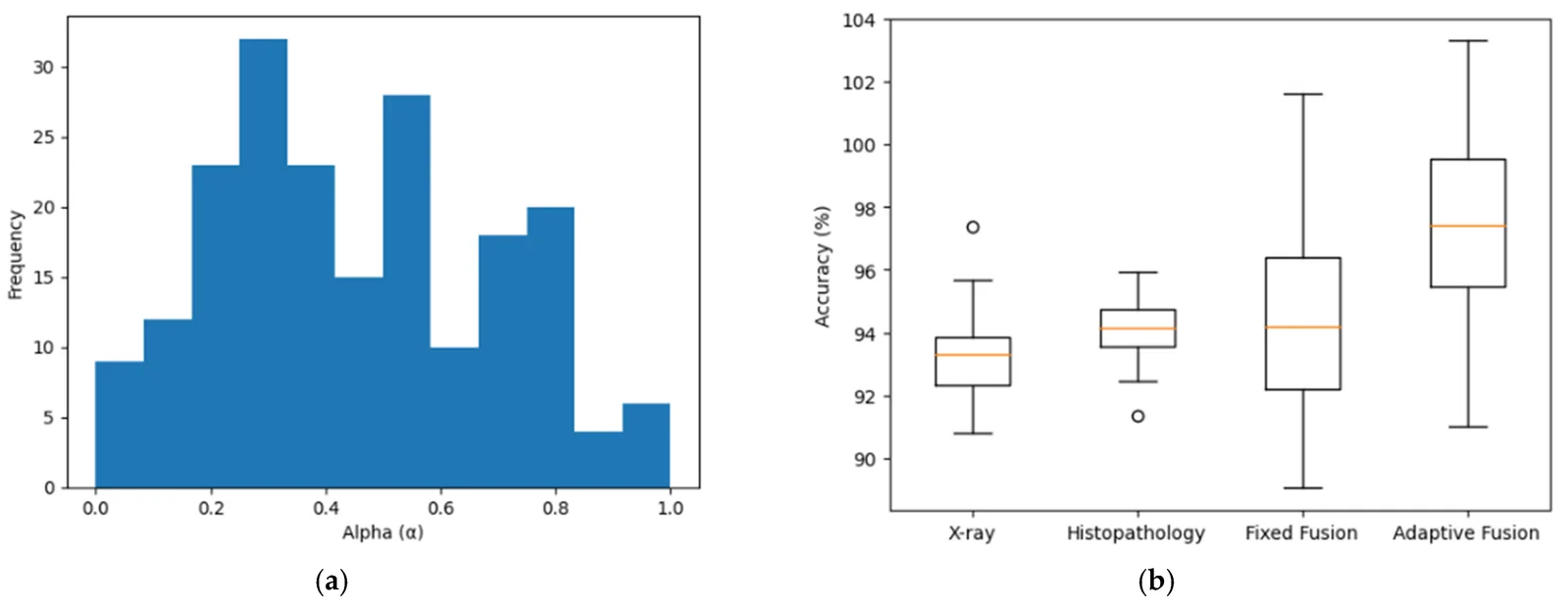
Distribution of learned fusion weights and performance comparison. (**a**) Histogram of adaptive fusion weights (α) predicted by the gating network on the test set. The distribution spans a wide range, indicating that the model dynamically adjusts modality contributions rather than collapsing to a constant value. (**b**) Boxplot of classification accuracy across different models. The adaptive fusion method achieves the highest median accuracy with competitive variance, demonstrating both effectiveness and stability.

Overall, the results demonstrate that adaptive fusion yields a clear and stable improvement in performance over unimodal and fixed-fusion approaches under unpaired data conditions. Table 7 shows the performance comparison of unimodal and fusion models. To further investigate the behavior of the adaptive fusion mechanism, we analyze the distribution of the learned fusion weights (α) and compare performance variability across different fusion strategies. The results are shown in Figure 5.

### 4.6. Explainability Analysis

**Figure 6:**
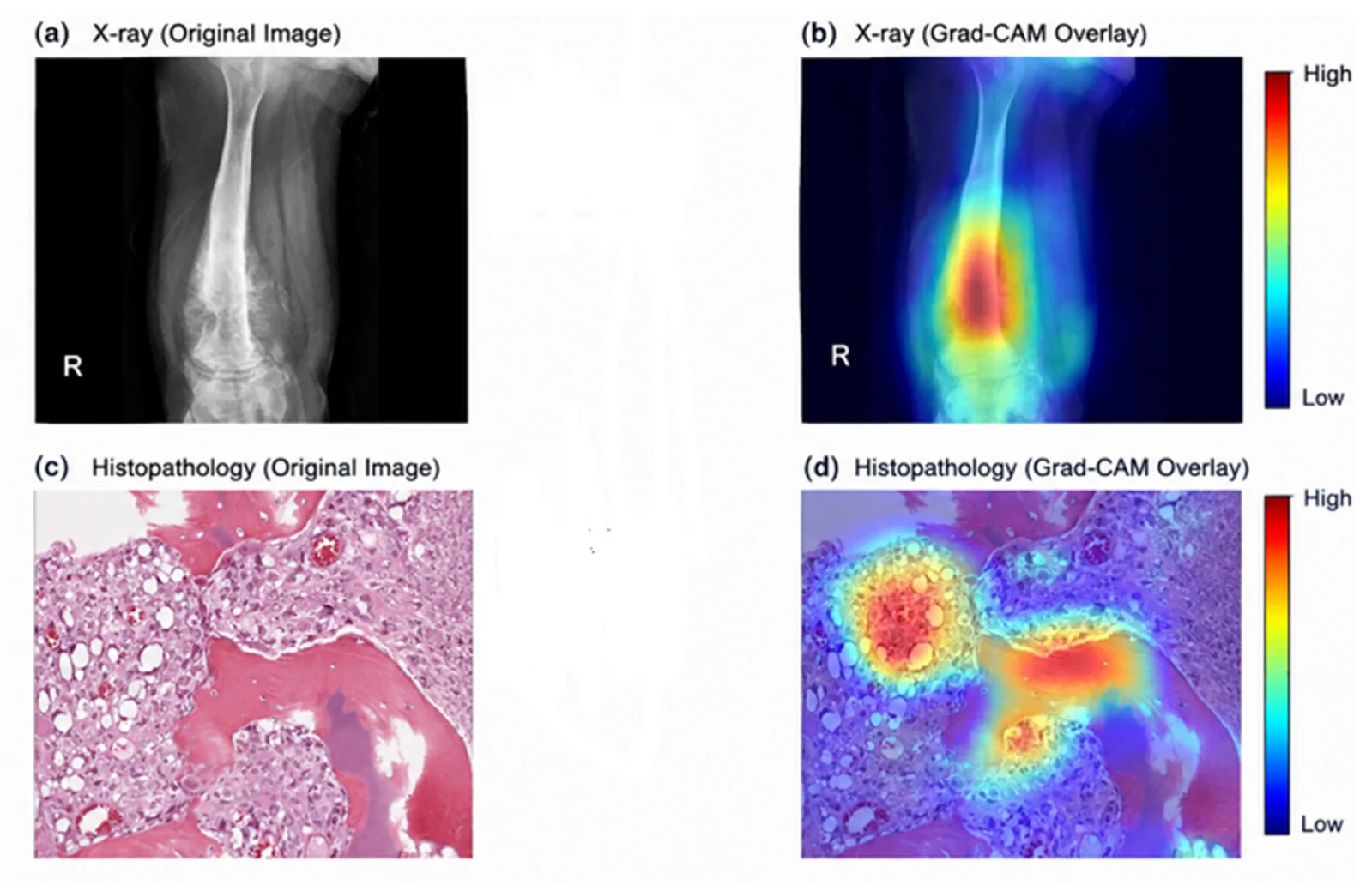
Grad-CAM visualization of model predictions for X-ray (**a**,**b**) and histopathology (**c**,**d**) images. Warmer colors indicate regions with higher contribution to the prediction. R refers to the right femur.

To improve model interpretability, Grad-CAM was used to visualize the regions contributing to the predictions of the X-ray and histopathology EfficientNet-B0 models. As shown in Figure 6, the X-ray visualization highlights the lesion-containing region, while the histopathology visualization emphasizes tumor-associated tissue regions. Warmer colors indicate higher relative contribution to the model prediction. These visualizations provide qualitative insight into the model’s decision-making.

## 5. Discussion

The experimental results demonstrate that integrating independently trained X-ray and histopathology models through decision-level fusion can effectively improve osteosarcoma classification performance under unpaired data conditions. Unlike conventional multimodal approaches that rely on feature-level alignment or patient-level correspondence, the proposed framework operates in a modality-agnostic manner, where each modality contributes independently through probability-level outputs.

The performance difference between fixed-weight fusion and adaptive gating fusion highlights the importance of sample-dependent modality contribution. Fixed fusion assumes a constant contribution ratio between X-ray and histopathology across all samples, which is not suitable for medical imaging scenarios where the discriminative strength of each modality varies significantly. In contrast, the gating network learns a sample-dependent weighting function from the modality probability distributions; therefore, the learned α should be interpreted as a confidence-driven fusion weight rather than a direct measure of clinical reliability. This adaptive behavior explains the consistent reduction in misclassifications observed in the confusion matrices. In particular, the gating network reduces errors in cases where one modality is uncertain or less informative, by dynamically shifting reliance toward the stronger predictive source. This reduces modality dominance bias and improves class-wise balance, especially in the non-viable tumor category, where visual characteristics can be ambiguous in one modality but clearer in the other.

From a methodological perspective, the results indicate that decision-level adaptive fusion is a stable and effective strategy for integrating heterogeneous medical data when alignment between modalities is not available. The improvement over fixed fusion is not only reflected in overall accuracy but also in reduced variance across experiments, suggesting improved robustness under different initializations.

However, the proposed framework also has limitations. First, although it avoids the need for paired datasets, it does not perform patient-level multimodal inference, since X-ray and histopathology samples originate from independent cohorts. This limits direct clinical interpretability in real-world diagnostic workflows. Second, the absence of external multicenter validation restricts conclusions about generalization across different imaging protocols, scanners, and population distributions. Third, an important limitation is that the proposed fusion does not represent patient-level multimodal inference because the X-ray and histopathology datasets are independently collected and contain no one-to-one patient correspondence. The method therefore evaluates probability-level fusion of independently generated modality predictions rather than combining paired examinations from the same patient. In addition, the conversion of the binary X-ray output into three classes uses histopathology-derived class proportions, introducing a dependency between the modality representations.

Recent multimodal learning studies have explored attention-based, transformer-based, and gated fusion mechanisms for combining heterogeneous medical imaging modalities. These approaches generally learn joint representations or cross-modal interactions between modalities. In contrast, the present study focuses on decision-level fusion, where independently generated modality-specific probabilities are combined through a lightweight adaptive gating mechanism. This design is particularly suitable for the unpaired setting considered here, because it does not require image-level or patient-level correspondence between the two modalities.

In addition, while the adaptive gating mechanism improves performance, it remains dependent on probability-level inputs from independently trained models. This means that errors in individual modalities can still propagate into the fusion stage, especially when both modalities produce low-confidence predictions.

Overall, the findings support the hypothesis that modality-agnostic ensemble learning with adaptive decision-level fusion can effectively leverage complementary diagnostic information in unpaired medical datasets. The proposed framework provides a flexible alternative to traditional multimodal learning approaches and demonstrates strong potential for scenarios where data pairing is unavailable or impractical.

The proposed framework was primarily evaluated using EfficientNet-B0 as the feature extraction backbone. Although the fusion mechanism is formulated independently at the probability level and is therefore conceptually applicable to other architectures, a comprehensive comparison across multiple backbone families was beyond the computational scope of the present study. Future work will evaluate the proposed adaptive fusion strategy using additional CNN and transformer-based backbones to further assess its architecture independence.

Also, future work will focus on extending the framework to cross-institutional datasets, incorporating additional imaging modalities such as MRI, and exploring attention-based or transformer-driven fusion mechanisms to further enhance adaptability and generalization.

## 6. Conclusions and Future Work

This study proposed a modality-agnostic ensemble learning framework for osteosarcoma classification using independently collected X-ray and histopathology datasets. Unlike conventional multimodal approaches that rely on patient-level correspondence or paired data, the proposed framework operates under unpaired conditions by independently modeling each modality and performing decision-level fusion.

Two fusion strategies were investigated: fixed-weight probability fusion and an adaptive sample-level gating mechanism. The adaptive approach dynamically learns fusion weights based on modality-specific predictive distributions, enabling more flexible and robust integration of heterogeneous information. Experimental results demonstrate that the adaptive fusion strategy consistently outperforms fixed-weight fusion and unimodal baselines, achieving a maximum accuracy of 97.08%.

These findings indicate that adaptive decision-level fusion can effectively exploit complementary information from independently sourced medical imaging modalities, even in the absence of direct correspondence. The proposed framework provides a practical alternative for real-world clinical scenarios where multimodal data are fragmented across institutions or acquisition pipelines.

A limitation of this work is the absence of cross-institution external validation and patient-level multimodal pairing, which restricts direct clinical deployment. Future work will focus on validating the proposed framework on multicenter datasets and exploring more advanced fusion mechanisms, such as attention-based and transformer-driven architectures, to further enhance robustness and generalization.

## Figures and Tables

**Table 1:** Summary of related works on osteosarcoma analysis.

Ref	Dataset	Model	Modularity	Fusion Type	Evaluation Level	Limitations
[5], 2024	Institutional X-rays	Deep learning (DL)	Single	No Fusion	Patient level not validated	Limited diagnosis for ambiguous radiographic cases because only radiographs were used, since no histology or clinical integration
[6], 2025	Multicenter radiographs	Ensemble DL model	Single	No Fusion	Patient level not validated	Still reliant on X-rays; no CT, MRI, or histology; deployment viability is reduced by ensemble complexity
[7], 2025	TCIA	Hybrid CNN–ViT	Single	No Fusion	Patch-level	Patient-level diagnoses may not be accurately reflected by patch-level labeling; slide-level data leakage could occur; and the model may overfit.
[8], 2020	CT + MRI scans (institutional)	CT/MRI image fusion for 3D tumor margin assessment	Multimodal	Fixed	Patient-Level	Fixed fusion; lacks adaptability
[9], 2025	CT/MRI + histopathology (from multicenter)	Multimodal DL fusion	Multimodal	Fixed	Patient-Level	Fusion static rather than sample-adaptive; limited sample size; performance might not be cross-center generalizable
[10], 2024	Skin lesion datasets (ISIC dermoscopy + clinical metadata)	Multi-stage multimodal adaptive fusion	Multimodal	Adaptive	Patient-Level	Not examined for osteosarcoma; adaptive logic in radiology/histology fusion has not been shown; it might need large, annotated datasets;
[11], 2024	Multi-parametric prostate MRI	Adaptive dual-view MRI fusion	Multimodal	Adaptive	Patient-Level	Limited external validation; computationally intensive; generalizability to other centers untested
[12], 2026	Public oral cancer histopathology dataset; 1224 H&E images at 100× and 400× magnification, re-annotated into four diagnostic categories	Explainable MMX-ViT: multi-scale CNN + Vision Transformer with Adaptive Cross-Fusion Module (ACFM)	Single	Adaptive	Patient-Level	Limited external/multi-institutional validation; explainability needs pathologist-in-the-loop quantitative validation; no genomic/clinical multimodal integration
[13], 2026	Public CRC-100K/CRC-VAL-HE-7K-based colorectal histopathology dataset; H&E images covering nine tissue classes	EfficientNet-B3 with Hybrid Loss (Hybrid-B3) combining weighted cross-entropy and focal loss	Single	No Fusion	Image/Patch-Level	No patient-level or external clinical validation; benchmark histopathology data may not capture multicenter variation; no multimodal integration
[14], 2026	Multimodal breast cancer datasets: METABRIC, GSE2034, GSE2990, BCSC, and Breast Cancer Coimbra; clinical, molecular, demographic, and lifestyle data	Unified multimodal Transformer with survival and attention-guided classification modules	Multimodal	Adaptive/cross-modal feature fusion	Patient-Level	Not designed for osteosarcoma; relies primarily on non-imaging clinical/molecular modalities; applicability of the framework to radiology–histopathology fusion and osteosarcoma remains unvalidated; external generalizability across other cancer types requires further investigation

**Table 2:** Experimental parameters.

Parameter	Values Evaluated	Selected
Learning rate	1 × 10^−4^, 5 × 10^−4^, 1 × 10^−3^	1 × 10^−3^
Dropout rate	0.3, 0.5	0.5
Hidden units	8–16, 16–8, 32–16	16–8
A	0, 0.25, 0.5, 0.75, 1	0.25
Input image size	224 × 224	224 × 224
Model	EfficientNet-B0	EfficientNet-B0
Batch size	16, 32	32
Optimizer	Adam	Adam
Loss function	cross-entropy loss	cross-entropy loss
Epochs	30	30

**Table 3:** Gating network parameters.

Parameter	Value
Input	6-dimensional vector [, p_h]
Hidden Layer 1	16 units, ReLU activation
Hidden Layer 2	8 units, ReLU activation
Output Layer	1 unit, sigmoid activation (to constrain α ∈ [0,1])
Dropout	0.3 after each hidden layer
Regularization	L2 penalty = 1 × 10^−4^
Optimizer	Adam
Learning rate	1 × 10^−3^
Loss	MSE (Mean Squared Error) between predicted α and pseudo-labels α*
Epochs	10 with early stopping

**Table 4:** Evaluation metrics for fixed and adaptive fusion strategies (mean ± standard deviation).

**(A) Fixed-α Fusion (Overall Accuracy = 94.15 ± 3.55%)**				
**Class**	**Precision (%)**	**Recall (%)**	**F1-Score(%)**	**Specificity (%)**
Non-tumor	93.90 ± 7.9	96.25 ± 6.0	95.06 ± 5.9	94.51 ± 7.3
Viable tumor	92.45 ± 9.9	94.23 ± 9.9	93.33 ± 7.7	96.64 ± 6.0
Non-viable tumor	97.22 ± 8.5	89.74 ± 12.0	93.33 ± 9.0	99.24 ± 3.7
**(B) Adaptive Gating Fusion (Overall Accuracy = 97.08 ± 2.85%)**				
**Class**	**Precision (%)**	**Recall (%)**	**F1-Score (%)**	**Specificity (%)**
Non-tumor	97.50 ± 6.6	97.50 ± 4.2	97.50 ± 4.8	97.80 ± 6.1
Viable tumor	96.23 ± 6.0	98.08 ± 6.0	97.14 ± 4.8	97.8 ± 5.7
Non-viable tumor	97.37 ± 8.2	94.87 ± 9.6	96.10 ± 6.8	99.24 ± 3.7

**Table 5:** Three-class confusion matrices for fixed-α and adaptive fusion on the TCIA evaluation set.

**(A) Fixed-α Fusion**			
**Actual\Predicted**	**Non-Tumor**	**Viable Tumor**	**Non-Viable Tumor**
Non-tumor (80)	77	2	1
Viable tumor (52)	3	49	0
Non-viable tumor (39)	2	2	35
**(B) Adaptive Gating Fusion**			
**Actual\Predicted**	**Non-Tumor**	**Viable Tumor**	**Non-Viable Tumor**
Non-tumor (80)	78	1	1
Viable tumor (52)	1	51	0
Non-viable tumor (39)	1	1	37

**Table 6:** Comparative Performance of Osteosarcoma Classification Models.

Reference	Modularity	Dataset	Architecture	Accuracy
[19], 2020	Single	TCIA	Transfer learning with pre-trained CNNs	95.65%
[20], 2021	Single	TCIA	2 CNN models	86.00%
[21], 2023	Single	TCIA	MobileNetV2	91.00%
[22], 2024	Multimodal [Paired] (CT + demographics + genetics)	CT scans + demographics + genetic markers (for 225 patients from two hospitals)	PCA + Improved Particle Swarm Optimization for feature selection, Classifier: ensemble (ECOC-SVM)	80.00%
[23], 2024	Single	TCIA	New feature extractor Framework + XGBoost	96.84%
[24], 2024	Single	TCIA	VGG19	96.27%
[25], 2025	Single	Radiology images for 200 collected patients	region-based CNN	96.00%
[26], 2025	Multimodal [Paired] (HSI + H&E pathology)	hyperspectral imaging (HSI) + H&E-stained pathology	MobileNetV2 (spatial) + 1D-CNN (spectral) + self-attention for fusion	87.33%
Proposed fixed fusion	[Unpaired] (X-ray + Histopathology)	TCIA + BTXRD	EfficientNet-B0	94.15%
Proposed adaptive fusion	[Unpaired] (X-ray + Histopathology)	TCIA + BTXRD	EfficientNet-B0	97.08%

Note: It is important to highlight that, unlike most approaches that rely on paired patient-level data, the proposed framework operates on unpaired datasets, which introduces fundamental differences in data representation and limits direct comparability.

**Table 7:** Performance Comparison of Unimodal and Fusion Models.

Model	Accuracy (%)
X-ray Only	93.07 ± 1.12
Histopathology Only	94.12 ± 0.85
Fixed Fusion	94.15 ± 3.55
Random α	93.60 ± 3.20
Adaptive Fusion	97.08 ± 2.85

Note: The X-ray-only result is a binary BTXRD evaluation, whereas the histopathology-only and fusion results are three-class evaluations on the TCIA-based fusion setting.

## Data Availability

The datasets analyzed during the current study are publicly available: The Cancer Imaging Archive (TCIA): https://www.cancerimagingarchive.net/ (accessed on 30 September 2026). BTXRD Database: https://figshare.com/articles/dataset/A_Radiograph_Dataset_for_the_Classification_Localization_and_Segmentation_of_Primary_Bone_Tumors/27865398?file=50653575 (accessed on 30 September 2026) or: https://doi.org/10.6084/m9.figshare.27865398 (accessed on 30 September 2026). Additional materials are available from the corresponding author upon reasonable request.
